# The Presence of a CMV Immunodominant Allele in the Recipient Is Associated With Increased Survival in CMV Positive Patients Undergoing Haploidentical Hematopoietic Stem Cell Transplantation

**DOI:** 10.3389/fonc.2019.00888

**Published:** 2019-09-17

**Authors:** Dolores Grosso, Benjamin Leiby, Matthew Carabasi, Joanne Filicko-O'Hara, Sameh Gaballa, William O'Hara, John L. Wagner, Neal Flomenberg

**Affiliations:** ^1^Blood and Marrow Transplant Program, The Sidney Kimmel Cancer Center at Thomas Jefferson University, Philadelphia, PA, United States; ^2^Pharmacology and Experimental Therapeutics, The Sidney Kimmel Cancer Center at Thomas Jefferson University, Philadelphia, PA, United States

**Keywords:** CMV, immunodominant allele, haploidentical, transplant outcomes, graft versus host disease, DNA terminase complex inhibitor

## Abstract

Specific major histocompatibility (MHC) class I alleles dominate anti-CMV responses in a hierarchal manner. These CMV immunodominant (IMD) alleles are associated with a higher magnitude and frequency of cytotoxic lymphocyte responses as compared to other human leukocyte antigen (HLA) alleles. CMV reactivation has been associated with an increased incidence of graft-vs.-host disease and non-relapse mortality, as well as protection from relapse in HLA-matched HSCT settings. Less is known about the impact of CMV reactivation on these major outcomes after haploidentical (HI) HSCT, an increasingly applied therapeutic option. In HI HSCT, the efficiency of the immune response is decreased due to the immune suppression required to cross the MHC barrier as well as MHC mismatch between presenting and responding cells. We hypothesized that the presence of a CMV IMD allele would increase the efficiency of CMV responses after HI HSCT potentially impacting CMV-related outcomes. In this retrospective, multivariable review of 216 HI HSCT patients, we found that CMV+ recipients possessing at least 1 of 5 identified CMV IMD alleles had a lower hazard of death (HR = 0.40, *p* = 0.003) compared to CMV+ recipients not possessing a CMV IMD allele, and an overall survival rate similar to their CMV– counterparts. The analysis delineated subgroups within the CMV+ population at greater risk for death due to CMV reactivation.

## Introduction

CMV reactivation has been associated with protection from relapse in human leukocyte antigen (HLA) matched related and unrelated hematopoietic stem cell transplant (HSCT) settings (1–3), most pronounced in patients with acute myeloid leukemia (AML) (4). However, in many series, benefits derived from CMV reactivation in terms of relapse are outweighed by accompanying increases in non-relapse mortality (NRM) (1, 5) and death (5, 6). Herpesviruses such as CMV share peptide sequence homology with humans resulting in cross-reactive cytotoxic lymphocyte (CTL) responses to both the virus and self, providing an explanation as to why CMV has been associated with graft vs. host disease (GVHD) and protection from relapse (7–9).

After haploidentical HSCT (HI HSCT), CMV reactivation rates are higher than typically seen following HLA matched HSCT (10–13), in part due to immune suppression of donor effector cells necessary to safely cross the major histocompatibility complex (MHC) barrier (14). However, HI HSCT also represents a situation where CMV epitope presentation to responder cells occurs in the context of MHC mismatch, which may further decrease the efficacy of the immune response to CMV (15).

Robust reconstitution of CMV-specific CTLs (16), adoptive transfer of CMV-specific T cells (17), and aggressive pharmacological approaches to CMV prophylaxis (18) have been shown to reduce CMV reactivation and disease post HI HSCT. However, there is limited information regarding the impact of CMV reactivation on outcomes such as relapse incidence and overall survival (OS) after HI HSCT. In a small analysis of 36 HI HSCT recipients, Lin et al. (19) identified a protective effect of CMV reactivation on relapse in AML subjects which was offset by increased NRM. In HI HSCT regimens using cyclophosphamide (CY) tolerization as GVHD prophylaxis, CMV reactivation was associated with increased NRM in one analysis (20) but a causal relationship between CMV reactivation and any major transplant outcome was not found in two others (12, 21, 22). Thus, despite a high reactivation rate after HI HSCT, a clear pattern of relapse protection or survival differences due to CMV has not emerged at least in transplants using CY tolerization approaches.

Multiple MHC alleles are capable of presenting CMV antigens to CTLs (23), however a hierarchal functionality exists (24–28) such that specific MHC class I alleles dominate CMV responses preferentially presenting CMV epitopes even in the presence of other HLA alleles capable of doing so.

HLA A^*^01:01 (25, 29), A^*^02:01 (24, 26, 28), B^*^07:02 (24, 28, 30, 31), B^*^08:01(28, 32), and C^*^07:02 (27, 33) are amongst the most immunodominant (IMD) CMV alleles presenting pp65, immediate early 1 and pp50 CMV epitopes which have been identified as the most immunogenic of CMV proteins (28, 34–36). In addition to hierarchal dominance, these alleles have been associated with eliciting a higher response frequency of CMV-specific CTLs (24–26, 29, 37), a greater magnitude of CTL expansion upon stimulation (24, 26), and a more stable and stronger binding affinity with responding CMV-specific CTLs (30, 32).

Because CMV IMD alleles are associated with greater efficiency and CTL frequencies in CMV responses, we previously hypothesized that in HI HSCT, the expression of a CMV IMD allele by the donor, the recipient, or a match of a CMV IMD allele on the shared haplotype could strengthen anti-CMV responses in patients in which these alleles were present. Examination of CMV-related outcomes such as relapse, NRM, and OS based on the presence or absence of CMV IMD alleles would potentially help delineate recipient CMV risks and clarify the effects of CMV reactivation in HI HSCT.

A preliminary univariate analysis performed at our institution (38) showed that CMV reactivation rates and CMV copy number were not significantly influenced post HI HSCT by the presence or the matching of CMV IMD alleles. However, the expression of one or more CMV IMD alleles in the recipient only was associated with lower rates of NRM and relapse, as well as higher OS as compared to recipients without a CMV IMD allele.

## Materials and Methods

The objective of this single institution, retrospective, multivariable analysis was to confirm a beneficial impact of recipient CMV IMD allele positivity (CMV IMD+) on outcomes post HI HSCT.

There were three testing groups. The primary group consisted of CMV positive (CMV+) recipients expressing one or more CMV IMD alleles. The outcomes of this group were compared to both CMV+ recipients not possessing a CMV IMD allele and CMV negative (CMV–) recipients, none of whom had evidence of a primary CMV infection after HI HSCT. Comparison to the second group allowed an analysis of the impact of CMV IMD alleles in the setting of CMV reactivation which occurred in the majority of CMV+ patients. CMV– recipients were also used as a comparator group to confirm that the effects of CMV IMD alleles were specific to the setting of CMV reactivation.

**Figure 1:**
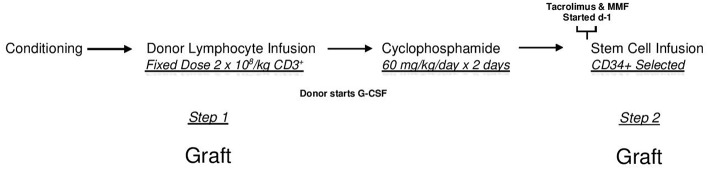
The two step approach. After conditioning all patients receive an unmanipulated donor product containing a fixed dose of 2 × 10^8^/kg donor CD3^+^ cells (DLI-step 1 of transplant). Two days after the DLI, Cyclophosphamide is infused at a dose of 60 mg/kg/day × 2 days for bidirectional tolerization of lymphocytes. After a day of rest, a CD34 selected donor stem cell product is infused (step 2 of transplant). Conditioning regimens were myeloablative consisting of 12 Gy total body irradiation (*n* = 128); reduced intensity total of 78 patients consisting of Fludarabine, 2 Gy total body irradiation, plus Cytarabine (*n* = 13) or Thiotepa (*n* = 45) or Busulfan (*n* = 20); or non-myeloablative (Fludarabine plus 2 Gy total body irradiation (*n* = 10). In this regimen, patients receive identical tolerized T cell doses and graft vs. host disease prophylaxis. Lymphocyte polarization is avoided as donors begin growth factors after lymphocyte collection.

All patients underwent HI HSCT on any of the Institutional Review Board-approved two-step research studies at Thomas Jefferson University Hospital between 2006 and 2019. The two step approach is shown in Figure 1. In this regimen, fixed dosing of CD3^+^ cells and a consistent GVHD prophylaxis approach provides a consistent platform to compare treatment effects amongst groups. Written consent was obtained from all patients in accordance with the Declaration of Helsinki.

HSCT outcomes examined for these groups were relapse, incidences of acute (aGVHD) and chronic (cGVHD), NRM, and OS. Confounders considered in the analysis were patient and donor age, paternal recipient based on a consistent finding of better outcomes in this patient group at our institution (39), conditioning intensity, myeloid vs. other diagnoses, Revised Disease Risk Index (RDRI) (40), CMV reactivation in the first 100 days post HSCT, Hematopoietic Cell Transplantation-Specific Comorbidity Index (41), the use of steroids within the first 100 days of HSCT, the presence of a killer immunoglobulin-like receptor (KIR) mismatch as defined by Ruggeri et al. (42), and donor B haplotype (43). Race/ethnicity (Race) was also considered as a confounder in the model to exclude the possibility that CMV IMD alleles, most commonly associated with Caucasians of European ancestry, were acting as a surrogate for the effect of Caucasian race on outcomes. Race has been found to have significant impacts on post HSCT outcomes in some analyses (44, 45).

To be included in the analysis, patients had to have had successful donor engraftment and no evidence of disease on the day +28 post marrow studies. Every patient transplanted on a two step HI HSCT study was included in this analysis if they met these criteria. Patients had lymphoid or myeloid malignancies. Five patients with aplastic anemia were included in the univariable analyses only for GVHD and OS.

### Statistical Analysis

Comparison of groups was performed using Fisher's exact test for categorical variables and ANOVA for continuous variables. For the outcomes analysis, univariate association of recipient characteristics with endpoints was evaluated using the competing risk method of Gray (46) (for relapse, NRM, aGVHD, and cGVHD) or the log rank test (OS). Analysis of aGVHD was censored at 180 days. Multivariable competing risk proportional hazards regression used the method of Fine and Gray (47). Cox proportional hazards regression was used for multivariable models of OS. NRM was the competing event for relapse, relapse was the competing event for NRM, and death was the competing event for aGVHD and cGVHD. Each endpoint was evaluated separately in CMV+ and CMV– recipients. Multivariable models assessed the association of CMV IMD allele with each outcome adjusted for recipient and donor age, paternal recipient, RDRI (categorized as High vs. Low), diagnosis, conditioning regimen, and race. Race was analyzed as a three category variable (Caucasian/African American/Other Minority) with type 3 *p*-values reported for this variable. Models for relapse, NRM, cGVHD and OS were also adjusted for steroid administration within 100 days and CMV reactivation within 100 days (CMV+ sample only). All analyses were completed using SAS version 9.4 (SAS Institute, Cary, NC).

### Definitions

CMV reactivation was defined as a positive result of >100 IU/ml on quantitative, real-time PCR testing of plasma within 100 days of HSCT. Preemptive therapy with valganciclovir was instituted for CMV reactivation. CMV IMD alleles were identified as HLA A^*^01:01, A^*^02:01, B^*^07:02, B^*^08:01, and C^*^07:02. aGVHD (grades 2–4) and cGVHD were identified and scored based on Glucksberg et al. (48) and National Institutes of Health Consensus Criteria, respectively (49). Relapse was defined as morphologic recurrence of disease. NRM was defined as death from any cause while the underlying malignancy was in remission. AML, myelodysplastic syndrome, chronic myelomonocytic leukemia, and myeloproliferative disorder were defined as myeloid malignancies.

## Results

### Patients

The outcomes of 216 consecutive two-step patients meeting inclusion criteria were analyzed. Supplementary Tables 1, 2 contain complete details of the analyses. There were 127 CMV+ and 89 CMV– recipients. Median follow-up of the participants was 23.7 months. Median and mean peripheral blood donor T cell chimerism of the group was 100 and 99.3%, respectively. Patient characteristics and association of confounders with CMV IMD alleles are contained in Table 1. Included in the analysis were 150 Caucasians, 45 African Americans, and 21 patients characterized as “Other Minority” (Non-African America minorities: 9 Hispanic, 11 Asian, and 1 Multiple Race). As expected, Caucasians possessed a significantly higher percentage of CMV IMD alleles compared to non-Caucasians in both the CMV+ and CMV– groups.

**Table 1:** Associations of confounders with CMV IMD alleles.

	**CMV+** **patient group**			**CMV– patient group**		
	**IMD Allele+** ***n* = 75**	**IMD Allele–** ***n* = 52**	***P-*value**	**IMD Allele+ *n* = 63**	**IMD Allele–** ***n* = 26**	***P*-value**
**Patient median age (years)**	59 range 20–77	54 range 19–78	0.065	52 range 19–74	48 range 21–74	0.771
**Race**			<0.001			<0.001
Caucasian	54 (72%)	19 (36.5%)		59 (94%)	18 (69%)	
AA	16 (21%)	19 (36.5%)		4 (6.0%)	6 (23%)	
Other minority	5 (7%)	14 (27%)		0 (0)	2 (8%)	
**Paternal recipient**	25/75 (33%)	19/52 (37%)	0.710	20/63 (32%)	6/26 (23%)	0.455
**Conditioning**			0.370			0.053
MA	40 (53%)	32 (62%)		44 (70%)	12 (46%)	
RIC/NMA	35 (47%)	20 (38%)		19 (30%)	14 (54%)	
**Disease type**			1.00			0.264
Myeloid	44 (58.5%)	30 (58%)		38 (60%)	12 (46%)	
Lymphoid	29 (38.5%)	21 (40%)		23 (37%)	14 (54%)	
Aplastic Anemia	2 (3%)	1 (2%)		2 (3%)	0 (0)	
**RDRI**			0.115			0.789
Low	2 (3%)	3 (6%)		3 (5%)	2 (8%)	
Intermediate	38 (52%)	16 (31%)		28 (46%)	11 (42%)	
High	30 (41%)	29 (57%)		29 (47%)	12 (46%)	
Very High	3 (4%)	3 (6%)		1 (2%)	1 (4%)	
**CMV reactivation first 100 days**	60/75 (80%)	41/52 (80%)	0.874	0	0	N/A
**Median HCT-CI**	3 range 0–7	3 range 0–5	0.263	2 range 0–6	3 range 0–6	0.341
**Steroid use first 100 days**			0.852			1.00
Yes	28 (37%)	18 (35%)		26 (41%)	11 (42%)	
No	47 (63%)	34 (65%)		37 (59%)	15 (58%)	
**Any KIR mismatch**	25/75 (33%)	18/52 (35%)	0.52	28/63 (44%)	10/26 (38%)	0.645
**Donor B haplotype**	50/73 (68%)	30/49 (61%)	0.566	43/63 (68%)	19/26 (73%)	0.801
Neutral	31	15		24	8	
Better	10	10		15	7	
Best	9	5		4	4	
**Donor median age**	39 range 19–66	39 range 18–67	0.578	44 range 18–68	49 range 24–63	0.332
**Donor CMV status**			0.470			0.071
Positive	41 (55%)	32 (62%)		14 (22%)	11 (42%)	
Negative	34 (45%)	20 (38%)		49 (78%)	15 (58%)	

*AA, African American; KIR, killer cell immunoglobulin-like receptor; HCT-CI, hematopoietic cell transplantation-specific comorbidity index; MA, myeloablative; NMA, non-myeloablative; RDRI, revised disease risk index; RIC, reduced intensity conditioning*.

### CMV Serostatus, Reactivation, and Tissue Disease

In the total healthy donor population of 216 subjects, the presence of a CMV IMD allele predicted for a CMV negative serostatus. Fifty-two of 136 (38%) CMV IMD allele+ donors vs. 46/80 (58%) CMV IMD allele- donors were CMV+, Chi Square 0.007. The same trend occurred in the recipient group where 75/138 (54%) of CMV IMD allele+ vs. 52/78 (67%) of CMV IMD- recipients were CMV+ although these differences did not reach statistical significance, Chi Square *p* = 0.077.

In the CMV+ group, 101/127 (80%) of patients had laboratory evidence of CMV reactivation within the first 100 days of HI HSCT. There was no significant difference in CMV reactivation rates based on the presence or absence of a recipient CMV IMD allele, 80 vs. 79%, respectively, Chi-Square *p* = 0.87.

Nine of 127 (7%) patients in the CMV+ group developed tissue manifestations of CMV. In this group of 9 patients, 2/3 of patients were CMV IMD allele-, and 2/3 of patients had CMV– donors. In five patients, CMV tissue disease developed in the gut (4) and retina (1) between days 95 and 365 (median d +170) in the context of treatment with steroids for GVHD. In the remaining 4 patients, CMV tissue disease developed earlier in the gut (2) and lung (2), between days 33 and 52 (median d +48). All four of these earlier patients were CMV IMD allele- and 2/4 had CMV– donors. Only one of these four early patients was being treated for GVHD with steroids at the time of tissue disease. CMV pneumonitis was the primary cause of death in two patients-both in the early group. These patients were not being treated for GVHD at the time of tissue disease, were CMV IMD allele-, and one of the two patients had a CMV– donor.

CMV tissue disease was treated with foscarnet or ganciclovir, and in the cases of CMV pneumonitis, CMV-specific gamma globulin.

No patient in the CMV– group had evidence of primary CMV infection post HSCT.

### Relapse

**Figure 2:**
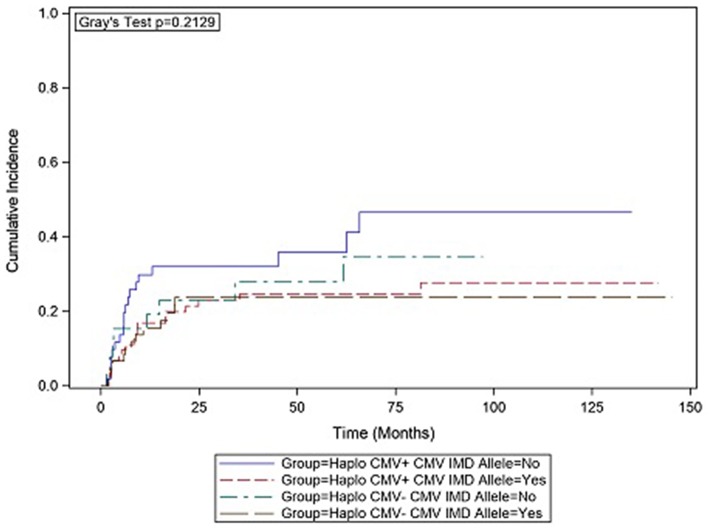
CMV+ patients without a CMV immunodominant allele had the highest cumulative incidence of relapse. although the results did not reach statistical significance.

In CMV+ recipients, the presence of a CMV IMD allele was associated with a lower hazard of relapse (HR = 0.47 *p* = 0.136) although in the current analysis, the result did not reach statistical significance. Cumulative incidence (CI) plot (Figure 2) shows that CMV+ recipients who lacked a CMV IMD allele had the highest relapse rate amongst the CMV+ and CMV– groups although this analysis also failed to reach statistical significance (*p* = 0.213). CI relapse in CMV+/IMD allele+ vs. CMV+/IMD allele- patients at 2 years was 23 vs. 32%, respectively. In the CMV+ group, a low RDRI score (HR = 0.44, *p* = 0.03) and paternal recipient status (HR = 0.25, *p* = 0.005) were associated with lower relapse rates.

### Acute GVHD

The presence or absence of a CMV IMD allele was not associated with aGVHD in either CMV+ or CMV– recipients. While not reaching statistical significance (*p* = 0.123), Caucasians were more likely to develop aGVHD in both the CMV+ and CMV– groups (HR = 2.13 and 3.42, respectively) as compared to African Americans.

### Chronic GVHD

cGVHD was not related to the presence or absence of a CMV IMD allele in CMV+ or CMV– recipients. In the CMV+ group, father recipient (HR = 3.57, *p* = 0.046) was associated with the development of cGVHD, while a high RDRI score was associated with a decreased hazard of cGVHD (HR = 0.20, *p* = 0.012). Race was associated with cGVHD (Type 3 *p* = 0.003) in the CMV+ group. Caucasians were less likely to develop cGVHD (HR = 0.201, *p* = 0.0078) and non-African American minorities had a relatively equal hazard of developing cGVHD (HR = 1.2, *p* = 0.82) as compared to African Americans. The CMV– group analysis for the impact of race on cGVHD was not able to be assessed due to the low number of non-African American minorities in this cohort.

### Non-relapse Mortality

The presence or absence of a CMV IMD allele was not significantly associated with NRM in any group. In CMV+ recipients, HR for NRM was 0.73 in CMV IMD allele+ vs. 1.37 in CMV IMD allele- recipients (*p* = 0.43). In CMV– recipients HR for NRM was 0.47 in CMV IMD allele+ vs. 2.137 in CMV IMD allele- recipients (*p* = 0.13) In CMV+ recipients, CMV reactivation (HR = 7.27, *p* = 0.057) and the use of steroids in the first 100 days (HR = 2.87, *p* = 0.015) were associated with an increase in NRM. Race was not associated with NRM.

### Survival

**Figure 3:**
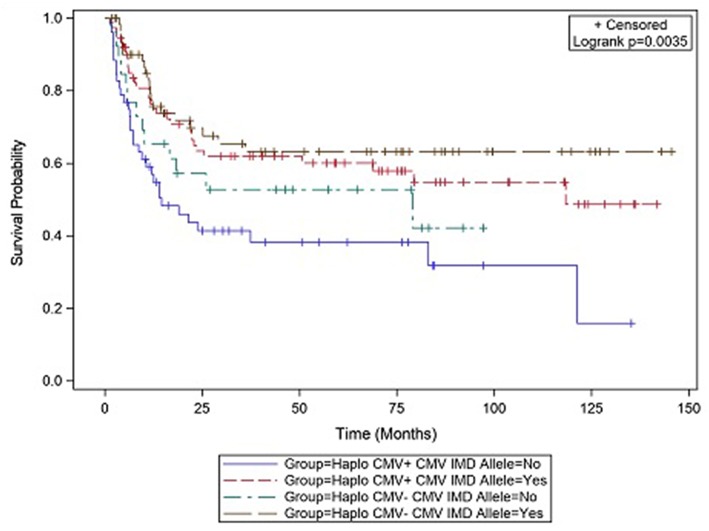
Survival differences in CMV+ vs. CMV– patients based on the presence or absence of a CMV immunodominant allele.

CMV reactivation was associated with increased mortality (HR = 3.42, *p* = 0.007). However, the risk of death was considerably lower in CMV+ recipients possessing a CMV IMD allele (HR = 0.40, *p* = 0.003) vs. those CMV+ recipients in which an allele was not present. As shown in the CI plot (Figure 3), the presence of a CMV IMD allele in CMV+ patients greatly increased OS compared to CMV+ recipients without the allele (Logrank *p* = 0.0035). CI of OS in CMV+/IMD allele+ vs. CMV+/IMD allele- patients at 2 years was 63 vs. 41%, respectively. CMV+ patients possessing a CMV IMD allele experienced OS curves that were similar to their CMV– counterparts. The presence of a CMV IMD allele was also associated with a lower hazard of death in CMV– recipients (HR = 0.58), although the result did not reach statistical significance (*p* = 0.18).

**Figure 4:**
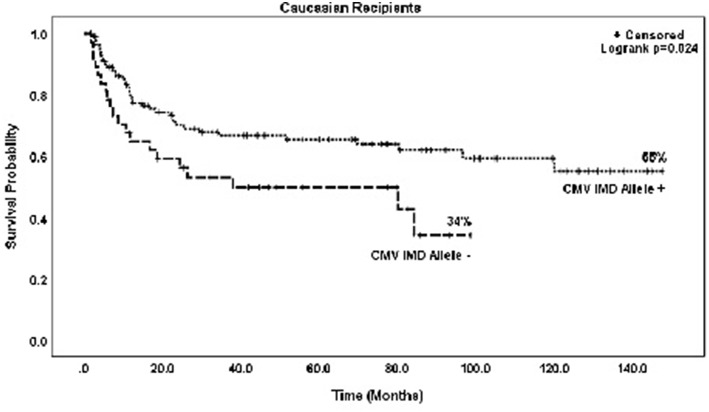
Probability of survival in CMV+ Caucasian patients based on the presence or absence of a CMV immunodominant allele.

In both groups, mortality was higher with increasing RDRI score reaching significance in the CMV+ group (HR = 1.97, *p* = 0.015). Risk of death was lower in recipients with myeloid vs. lymphoid diseases, reaching statistical significance in the CMV+ group (HR = 0.45, *p* = 0.011). There were no significant differences in risk of mortality based on race/ethnicity; Caucasian (HR = 0.92)and non-Caucasian non-African American (HR = 0.91, type 3 *p* = 0.956) vs. African American recipients. CMV+ Caucasian recipients not in possession of a CMV IMD allele had a significantly lower OS rate vs. CMV+ Caucasians with a CMV IMD allele on Kaplan Meier analysis, *p* = 0.024 (Figure 4).

## Discussion

The primary finding of this multivariable analysis was that the presence of a CMV IMD allele in CMV+ recipients undergoing HI HSCT significantly reduced the hazard of post-transplant death as compared to those not possessing a CMV IMD allele. Moreover, the possession of a CMV IMD allele served to increase the OS rate in CMV+ recipients, most of whom developed CMV reactivation, to that of CMV– recipients none of whom had evidence of CMV post HSCT. Accordingly, CMV+ recipients without a CMV IMD allele had the poorest CI of OS at <20%.

In contrast to our preliminary univariable analysis (38), the impact of CMV IMD alleles on relapse incidence and NRM in CMV+ recipients was not statistically significant in this multivariable study. Because the hazards for NRM and especially for relapse were lower in the CMV IMD allele+ group, it is possible that a combination effect from these factors was responsible for the OS benefit. However, CMV reactivation was significantly associated with increased NRM in CMV+ recipients, and reactivation occurred equally between CMV IMD allele+ and CMV IMD allele- groups. This finding suggests that the OS benefit associated with the possession of a CMV IMD allele was derived more substantively from relapse protection.

The preliminary analysis (38) as well as an internal multivariable analysis (Grosso, unpublished) showed that any benefits derived from the presence CMV IMD alleles were specific to the possession of one or more of these alleles by the recipient only. Responding CTLs in this analysis were of donor origin suggesting that the effects were due to recipient antigen presentation.

The ability to compensate for CMV immunoevasions (27, 50) and to present multiple CMV epitopes (8, 24) are characteristics of the CMV IMD alleles tested in this analysis. More efficient antigen presentation by host antigen presenting cells expressing a CMV IMD dominant allele or their ability to stimulate higher frequency donor CMV-specific CTL responses potentially strengthened a graft vs. tumor response resulting in a survival benefit. Testing of this hypothesis and the results of this analysis in general require further study.

There was no evidence that the presence of a CMV IMD allele was related to the occurrence of GVHD, nor was there evidence that the presence of a recipient CMV IMD allele affected HSCT outcomes, including OS, in the absence of CMV reactivation.

In some HI HSCT settings, natural killer (NK) effects such as KIR mismatch between the donor and the recipient or the presence of an activating KIR centromeric and telomeric B motif in the donor, are associated with lower relapse rates and improved OS aft HSCT (42, 43). Because there was an equal distribution of these characteristics between the CMV IMD positive and negative groups, we do not believe that OS differences in this analysis were due to NK effects.

In contrast to the strong association between CMV IMD alleles and improved survival, mortality differences between different races and ethnicities was not observed. This finding is inconsistent with CMV IMD alleles serving as surrogates for Caucasian race in this analysis. The finding that Caucasians not possessing CMV IMD alleles had lower OS rates than Caucasians with these alleles strengthens this conclusion. There was a highly significant relationship between non-Caucasians and an increased incidence of cGVHD in the current study. This finding is in agreement with Solomon et al. (51) in which African Americans undergoing post-transplant CY HI HSCT were found to have higher incidences of cGVHD, but in contrast to our analysis, lower rates of mortality and relapse as compared to Caucasians. There is limited information regarding the effect of race on outcomes post HI HSCT in general and this area requires continued investigation.

We opted to include the small percentage of CMV+ patients who had no microbiologic evidence of CMV reactivation post HI HSCT in the CMV+ group to avoid the loss of subjects with undetected, transient CMV reactivation. We have demonstrated large increases in CTL frequency after CMV reactivation in HI HSCT recipients (22, 52, 53). While the median d +90 CD3/8^+^ cell frequency of these CMV+ “non-reactivators” was lower than that of CMV+ patients reactivating CMV (Mann-Whitney *p* = 0.005), it was significantly higher than patients in the CMV– group (Mann-Whitney *p* = 0.019). This suggested that there was likely subclinical, undetected CMV reactivation in at least some subjects who were classified as not reactivating CMV justifying inclusion into CMV+ testing group.

Limitations of this study include its retrospective nature and specificity to the two step population. While the homogeneous nature of the two step HI HSCT approach reduces the confounders of varying T cell doses and GVHD prophylaxis strategies on the current results, a broader analyses of patient outcomes based on the presence or absence of CMV IMD alleles in the setting of alternate HI-HSCT approaches should be performed to confirm these results. *In vitro* studies specifically examining anti-CMV responses and potential anti-tumor effects of cross-reactive CMV-specific CTLs in patients possessing CMV alleles of varying immunodominance are planned at our institution to further explore these results.

In summary, the presence of a CMV IMD allele as defined in this study, was associated with protection from death in patients reactivating CMV after HI HSCT. To our knowledge, this is the first analysis of post HI HSCT outcomes based on the presence or absence of a CMV IMD allele. The data potentially delineates a high risk subpopulation of patients within the CMV+ group who do not benefit from a GVT effect related to CMV reactivation while simultaneously at risk for the negative effects of CMV reactivation. Consequently, the CMV IMD allele- group within the CMV+ population is the most likely to benefit from CMV prophylaxis with DNA terminase complex inhibitors. Another question raised by the data is whether the OS benefit associated with CMV reactivation in the CMV+/CMV IMD allele+ population justifies the withholding of DNA terminase complex inhibitors in this group. Additional analyses in larger groups of patients to assess whether the potential beneficial effects of CMV reactivation on both relapse and NRM outweigh deleterious effects in the CMV IMD allele+ subgroup. The results of this study support continued analysis in this area.

## Data Availability

The datasets generated for this study are available on request to the corresponding author.

## Ethics Statement

This studies involving human participants were reviewed and approved by Thomas Jefferson University Institutional Review Board. The patients/participants provided their written informed consent to participate in this study.

## Author Contributions

DG developed the research, interpreted the analysis, and was the primary manuscript author. BL performed the statistical analysis and assisted in interpreting the data. MC, JF-O'H, SG, WO'H, and JW assisted in the interpretation of the data, contributed to, and edited the manuscript. NF assisted in developing the research, analyzing results, and editing and approving of the manuscript.

### Conflict of Interest Statement

The authors declare that the research was conducted in the absence of any commercial or financial relationships that could be construed as a potential conflict of interest.
