# Supplementary material for: The Presence of a CMV Immunodominant Allele in the Recipient Is Associated With Increased Survival in CMV Positive Patients Undergoing Haploidentical Hematopoietic Stem Cell Transplantation
Source: Front Oncol. 2019 Sep 17;9:888. doi: 10.3389/fonc.2019.00888 (PMC6758597; doi:10.3389/fonc.2019.00888)
Supplement: Supplementary file 1 [file Table_1.docx]

**Supplementary Table 1.**

Univariable Regression Results

**Outcome=Relapse**

| **Variable** | **CMV Positive**  **HR (CI)**  **p-value** | **Type 3 p-value** | **CMV Negative**  **HR (CI)**  **p-value** | **Type 3 p-value** |
| --- | --- | --- | --- | --- |
| Age | 0.98 (0.96,1.01);  p= 0.150 | . | 1.00 (0.98,1.03);  p= 0.769 | . |
| CMV reactivation by Day 100 | 0.88 (0.41,1.89);  p= 0.744 | . |  | . |
| Donor Age | 1.02 (0.99,1.04);  p= 0.168 | . | 1.01 (0.98,1.04);  p= 0.510 | . |
| Myeloid Vs Lymphoid | 0.67 (0.34,1.31);  p= 0.244 | . | 0.52 (0.20,1.32);  p= 0.168 | . |
| RIC vs Myeloablative | 0.81 (0.43,1.53);  p= 0.514 | . | 0.55 (0.24,1.28);  p= 0.167 | . |
| Race/Ethnicity-Caucasian | 0.75 (0.37,1.52);  p= 0.422 | 0.6986 | 0.82 (0.25,2.68);  p= 0.738 | <.0001 |
| Race/Ethnicity-Other Minority | 0.74 (0.27,2.02);  p= 0.551 |  | 0.00 (0.00,0.00);  p= <.001 |  |
| CMV IMD Allele | 0.57 (0.30,1.09);  p= 0.090 | . | 0.71 (0.30,1.69);  p= 0.443 | . |
| Steroid Use During First 100 day | 0.64 (0.31,1.34);  p= 0.236 | . | 0.49 (0.19,1.23);  p= 0.127 | . |
| Dad Recipient | 0.35 (0.16,0.77);  p= 0.010 | . | 0.57 (0.19,1.69);  p= 0.309 | . |
| High RDRI | 2.28 (1.16,4.49);  p= 0.017 | . | 1.36 (0.59,3.17);  p= 0.470 | . |

**Outcome=Acute GVHD**

| **Variable** | **CMV Positive**  **HR (CI)**  **p-value** | **Type 3 p-value** | **CMV Negative**  **HR (CI)**  **p-value** | **Type 3 p-value** |
| --- | --- | --- | --- | --- |
| Age | 1.01 (0.99,1.03);  p= 0.505 | . | 0.99 (0.97,1.00);  p= 0.102 | . |
| Donor Age | 1.02 (1.00,1.04);  p= 0.023 | . | 1.01 (0.99,1.04);  p= 0.217 | . |
| Myeloid Vs Lymphoid/Aplastic Anemia | 2.57 (0.48,13.7);  p= 0.270 | 0.4835 | 1.06 (0.17,6.43);  p= 0.952 | 0.6297 |
| Myeloid Vs Lymphoid | 1.19 (0.70,2.03);  p= 0.527 |  | 1.34 (0.74,2.41);  p= 0.338 |  |
| RIC vs Myeloablative | 0.78 (0.46,1.31);  p= 0.351 | . | 1.20 (0.64,2.22);  p= 0.569 | . |
| Race/Ethnicity Caucasian | 2.34 (1.18,4.61);  p= 0.015 | 0.0393 | 2.28 (0.79,6.57);  p= 0.125 | 0.3000 |
| Race/Ethnicity Other Minority | 1.51 (0.64,3.57);  p= 0.352 |  | 2.90 (0.18,46.9);  p= 0.453 |  |
| CMV IMD Allele | 1.00 (0.60,1.67);  p= 0.994 | . | 0.92 (0.50,1.68);  p= 0.777 | . |
| Dad Recipient | 1.11 (0.66,1.88);  p= 0.691 | . | 0.58 (0.30,1.13);  p= 0.108 | . |
| High RDRI | 0.81 (0.48,1.37);  p= 0.426 | . | 0.79 (0.43,1.43);  p= 0.430 | . |

**Outcome=Chronic GVHD**

| **Variable** | **CMV Positive**  **HR (CI)**  **p-value** | **Type 3 p-value** | **CMV Negative**  **HR (CI)**  **p-value** | **Type 3 p-value** |
| --- | --- | --- | --- | --- |
| Age | 1.01 (0.98,1.04);  p= 0.636 | . | 1.00 (0.98,1.03); p= 0.771 | . |
| CMV reactivation by Day 100 | 0.57 (0.22,1.46);  p= 0.243 | . |  | . |
| Donor Age | 1.01 (0.98,1.04);  p= 0.584 | . | 0.99 (0.96,1.02); p= 0.648 | . |
| Myeloid Vs Lymphoid/Aplastic Anemia | 2.67 (0.26,27.8);  p= 0.411 | 0.6399 | 0.00 (0.00,0.00); p= <.001 | <.0001 |
| Myeloid Vs Lymphoid | 0.85 (0.34,2.14);  p= 0.734 |  | 0.78 (0.31,1.93); p= 0.587 |  |
| RIC vs Myeloablative | 1.85 (0.71,4.81);  p= 0.207 | . | 0.78 (0.32,1.94); p= 0.598 |  |
| Race/Ethnicity Caucasian | 0.46 (0.18,1.19);  p= 0.109 | 0.2293 | 0.39 (0.12,1.28);  p= 0.121 | <.0001 |
| Race/Ethnicity Other Minority | 0.97 (0.29,3.29);  p= 0.963 |  | 0.00 (0.00,0.00);  P= |  |
| CMV IMD Allele | 0.43 (0.18,1.05);  p= 0.063 | . | 0.98 (0.38,2.53);  p= 0.959 | . |
| Steroid Use During First 100 day | 1.44 (0.60,3.45);  p= 0.410 | . | 0.87 (0.36,2.12);  p= 0.761 | . |
| Dad Recipient | 1.94 (0.82,4.63);  p= 0.134 | . | 0.68 (0.22,2.08);  p= 0.496 | . |
| High RDRI | 0.61 (0.24,1.53);  p= 0.292 | . | 0.49 (0.19,1.31);  p= 0.155 | . |

**Outcome=Non-Relapse Mortality**

| **Variable** | **CMV Positive**  **HR (CI)**  **p-value** | **Type 3 p-value** | **CMV Negative**  **HR (CI)**  **p-value** | **Type 3 p-value** |
| --- | --- | --- | --- | --- |
| Age | 1.02 (0.99,1.05);  p= 0.129 | . | 1.02 (0.98,1.06);  p= 0.349 | . |
| CMV reactivation by Day 100 | 8.19 (1.20,55.8);  p= 0.032 | . |  | . |
| Donor Age | 0.98 (0.95,1.00);  p= 0.102 | . | 1.02 (0.99,1.05);  p= 0.309 | . |
| Myeloid Vs Lymphoid | 0.83 (0.40,1.70);  p= 0.602 | . | 0.88 (0.35,2.23);  p= 0.788 | . |
| RIC vs Myeloablative | 1.07 (0.53,2.15);  p= 0.856 | . | 0.72 (0.29,1.79);  p= 0.476 | . |
| Race/Ethnicity Caucasian | 0.56 (0.26,1.22);  p= 0.143 | 0.2217 | 1.13 (0.27,4.73);  p= 0.868 | <.0001 |
| Race/Ethnicity Other Minority | 1.10 (0.40,3.03);  p= 0.852 |  | 0.00 (0.00,0.00);  p= <.001 |  |
| CMV IMD Allele | 0.76 (0.38,1.53);  p= 0.442 | . | 0.53 (0.21,1.32);  p= 0.171 | . |
| Steroid Use During First 100 day | 1.34 (0.66,2.70);  p= 0.414 | . | 1.10 (0.44,2.76);  p= 0.836 | . |
| Dad Recipient | 2.25 (1.12,4.51);  p= 0.023 | . | 1.27 (0.49,3.32);  p= 0.625 | . |
| High RDRI | 1.25 (0.62,2.51);  p= 0.535 | . | 1.50 (0.60,3.75);  p= 0.383 | . |

**Outcome=Overall Survival**

| **Variable** | **CMV Positive**  **HR (CI)**  **p-value** | **Type 3 p-value** | **CMV Negative**  **HR (CI)**  **p-value** | **Type 3 p-value** |
| --- | --- | --- | --- | --- |
| Age | 1.00 (0.98,1.01);  p= 0.746 | . | 1.00 (0.98,1.02);  p= 0.902 | . |
| CMV reactivation by Day 100 | 0.87 (0.51,1.50);  p= 0.618 | . |  | . |
| Donor Age | 1.00 (0.98,1.02);  p= 0.659 | . | 1.01 (0.98,1.03);  p= 0.633 | . |
| Myeloid Vs Lymphoid/Aplastic Anemia | 1.96 (0.46,8.41);  p= 0.363 | 0.3173 | 0.91 (0.21,3.88);  p= 0.899 | 0.6905 |
| Myeloid Vs Lymphoid | 1.42 (0.86,2.37);  p= 0.173 |  | 1.25 (0.73,2.15);  p= 0.419 |  |
| RIC vs Myeloablative | 1.08 (0.66,1.79);  p= 0.754 | . | 0.76 (0.43,1.35);  p= 0.355 | . |
| Race/Ethnicity Caucasian | 0.80 (0.44,1.48);  p= 0.481 | 0.7165 | 0.56 (0.23,1.35);  p= 0.195 | 0.4265 |
| Race/Ethnicity Other Minority | 1.01 (0.40,2.56);  p= 0.989 |  | 0.67 (0.13,3.41);  p= 0.633 |  |
| CMV IMD Allele | 0.66 (0.38,1.14);  p= 0.133 | . | 0.78 (0.41,1.50);  p= 0.458 | . |
| Steroid Use During First 100 day | 0.90 (0.53,1.52);  p= 0.696 | . | 0.72 (0.42,1.24);  p= 0.235 | . |
| Dad Recipient | 1.18 (0.70,2.00);  p= 0.532 | . | 1.19 (0.66,2.14);  p= 0.559 | . |
| High RDRI | 1.33 (0.77,2.31);  p= 0.309 | . | 0.66 (0.37,1.18);  p= 0.163 | . |

HR, Hazard Ratio, CI, Confidence Interval

Race (Caucasian, African American, Other Minority) and Disease type (Myeloid Vs Lymphoid Vs Aplastic Anemia) were analyzed as 3 category variables and therefore type 3 p-values are reported for them. Only 2 patients had a diagnosis of aplastic anemia and only 2 patients were characterized as “Other Minority” in the CMV negative group. Therefore, Type 3 p-values specific to this CMV negative category were not reliably assessed. Patients with aplastic anemia were counted in graft versus host disease and survival analyses only.
