# Supplementary material for: The Presence of a CMV Immunodominant Allele in the Recipient Is Associated With Increased Survival in CMV Positive Patients Undergoing Haploidentical Hematopoietic Stem Cell Transplantation
Source: Front Oncol. 2019 Sep 17;9:888. doi: 10.3389/fonc.2019.00888 (PMC6758597; doi:10.3389/fonc.2019.00888)
Supplement: Supplementary file 2 [file Table_2.docx]

**Supplementary Table 2.**

Multivariable Regression Results

**Outcome=Relapse**

| **Variable** | **CMV Positive**  **HR (CI)**  **p-value** | **Type 3 p-value** | **CMV Negative**  **HR (CI)**  **p-value** | **Type 3 p-value** |
| --- | --- | --- | --- | --- |
| Age | 1.00 (0.97,1.02);  p= 0.756 |  | 0.99 (0.95,1.03);  p= 0.598 |  |
| CMV reactivation by Day 100 | 1.06 (0.43,2.62);  p= 0.898 |  |  |  |
| Donor Age | 0.99 (0.96,1.02);  p= 0.654 |  | 1.00 (0.95,1.05);  p= 0.972 |  |
| Myeloid Vs Lymphoid | 0.62 (0.29,1.31);  p= 0.211 |  | 0.49 (0.14,1.73);  p= 0.267 |  |
| RIC vs Myeloablative | 0.77 (0.38,1.53);  p= 0.452 |  | 0.57 (0.21,1.52);  p= 0.261 |  |
| Race/Ethnicity-Caucasian | 1.50 (0.52,4.30);  p= 0.454 | p=0.606 | 1.15 (0.30,4.41);  p= 0.842 | p= <.001 |
| Race/Ethnicity-Other Minority | 0.73 (0.24,2.22);  p= 0.584 |  | 0.00 (0.00,0.00);  p= <.001 |  |
| CMV IMD Allele | 0.47 (0.17,1.27);  p= 0.136 |  | 0.69 (0.24,1.94);  p= 0.481 |  |
| Steroid Use During First 100 days | 0.54 (0.26,1.14);  p= 0.105 |  | 0.51 (0.18,1.44);  p= 0.203 |  |
| Dad Recipient | 0.25 (0.09,0.66);  **p= 0.005** |  | 0.53 (0.09,3.11);  p= 0.481 |  |
| High RDRI | 2.29 (1.09,4.83);  **p= 0.029** |  | 1.38 (0.58,3.28);  p= 0.471 |  |

**Outcome=Acute GVHD**

| **Variable** | **CMV Positive**  **HR (CI)**  **p-value** | **Type 3 p-value** | **CMV Negative**  **HR (CI)**  **p-value** | **Type 3 p-value** |
| --- | --- | --- | --- | --- |
| Age | 1.01 (0.98,1.04);  p= 0.610 |  | 0.99 (0.96,1.01);  p= 0.291 |  |
| Donor Age | 1.02 (0.99,1.05);  p= 0.180 |  | 1.01 (0.98,1.05);  p= 0.509 |  |
| Myeloid Vs Lymphoid | 1.29 (0.70,2.38);  p= 0.409 |  | 0.94 (0.47,1.88);  p= 0.855 |  |
| RIC vs Myeloablative | 1.10 (0.52,2.31);  p= 0.803 |  | 1.23 (0.54,2.81);  p= 0.629 |  |
| Race/Ethnicity Caucasian | 2.13 (1.01,4.51);  p= 0.048 | p=0.123 | 3.42 (1.13,10.4);  p= 0.029 | p=0.098 |
| Race/Ethnicity Other Minority | 1.45 (0.56,3.76);  p= 0.446 |  | 1.58 (0.07,34.5);  p= 0.771 |  |
| CMV IMD Allele | 0.92 (0.51,1.66);  p= 0.782 |  | 0.73 (0.36,1.47);  p= 0.379 |  |
| Dad Recipient | 1.20 (0.50,2.92);  p= 0.681 |  | 0.88 (0.30,2.60);  p= 0.821 |  |
| High RDRI | 0.87 (0.49,1.55);  p= 0.637 |  | 0.58 (0.29,1.15);  p= 0.120 |  |

**Outcome=Chronic GVHD**

| **Variable** | **CMV Positive**  **HR (CI)**  **p-value** | **Type 3 p-value** | **CMV Negative**  **HR (CI)**  **p-value** | **Type 3 p-value** |
| --- | --- | --- | --- | --- |
| Age | 1.04 (1.00,1.09);  p= 0.074 |  | 1.01 (0.96,1.06);  p= 0.723 |  |
| CMV reactivation by Day 100 | 0.31 (0.09,1.11);  p= 0.071 |  |  |  |
| Donor Age | 1.03 (0.99,1.07);  p= 0.136 |  | 0.98 (0.94,1.02);  p= 0.348 |  |
| Myeloid Vs Lymphoid/Aplastic Anemia |  |  |  |  |
| Myeloid Vs Lymphoid | 0.93 (0.28,3.08);  p= 0.907 |  | 0.87 (0.29,2.56);  p= 0.793 |  |
| RIC vs Myeloablative | 3.08 (0.92,10.3);  p= 0.068 |  | 0.65 (0.18,2.34);  p= 0.511 |  |
| Race/Ethnicity Caucasian | 0.20 (0.06,0.66);  **p= 0.008** | p=0.003 | 0.37 (0.06,2.22);  p= 0.279 | p=<.0001 |
| Race/Ethnicity Other Minority | 1.21 (0.24,6.16);  p= 0.820 |  | 0.00 (0.00,0.00)  <.0001 |  |
| CMV IMD Allele | 0.45 (0.16,1.28);  p= 0.134 |  | 1.23 (0.42,3.58);  p= 0.704 |  |
| Steroid Use During First 100 days | 1.69 (0.58,4.89);  p= 0.336 |  | 1.12 (0.46,2.72);  p= 0.797 |  |
| Dad Recipient | 3.57 (1.02,12.5);  **p= 0.046** |  | 0.34 (0.06,1.81);  p= 0.206 |  |
| High RDRI | 0.20 (0.06,0.70);  **p= 0.012** |  | 0.67 (0.20,2.32);  p= 0.533 |  |

**Outcome=Non-Relapse Mortality**

| **Variable** | **CMV Positive**  **HR (CI)**  **p-value** | **Type 3 p-value** | **CMV Negative**  **HR (CI)**  **p-value** | **Type 3 p-value** |
| --- | --- | --- | --- | --- |
| Age | 1.03 (0.98,1.08);  p= 0.251 |  | 1.01 (0.97,1.06);  p= 0.559 |  |
| CMV reactivation by Day 100 | 7.27 (0.94,56.0);  **p= 0.057** |  |  |  |
| Donor Age | 0.98 (0.94,1.02);  p= 0.312 |  | 1.04 (0.99,1.09);  p= 0.152 |  |
| Myeloid Vs Lymphoid | 0.89 (0.40,1.98);  p= 0.777 |  | 1.09 (0.33,3.59);  p= 0.889 |  |
| RIC vs Myeloablative | 0.97 (0.45,2.08);  p= 0.940 |  | 1.07 (0.37,3.10);  p= 0.900 |  |
| Race/Ethnicity Caucasian | 0.42 (0.16,1.14);  p= 0.090 | p=0.228 | 1.43 (0.32,6.34);  p= 0.642 | p= <.001 |
| Race/Ethnicity Other Minority | 0.94 (0.30,2.93);  p= 0.909 |  | 0.00 (0.00,0.00);  p= <.001 |  |
| CMV IMD Allele | 0.73 (0.33,1.62);  p= 0.436 |  | 0.47 (0.17,1.26);  p= 0.132 |  |
| Steroid Use During First 100 days | 2.87 (1.23,6.70);  **p= 0.015** |  | 1.11 (0.42,2.90);  p= 0.834 |  |
| Dad Recipient | 1.85 (0.81,4.22);  p= 0.146 |  | 2.39 (0.44,13.0);  p= 0.314 |  |
| High RDRI | 1.27 (0.58,2.76);  p= 0.547 |  | 1.30 (0.44,3.81);  p= 0.633 |  |

Outcome=Overall Survival

| **Variable** | **CMV Positive**  **HR (CI)**  **p-value** | **Type 3 p-value** | **CMV Negative**  **HR (CI)**  **p-value** | **Type 3 p-value** |
| --- | --- | --- | --- | --- |
| CMV IMD Allele | 0.40 (0.22,0.73);  **p= 0.003** |  | 0.58 (0.26,1.29);  p= 0.185 |  |
| Age | 1.01 (0.99,1.04);  p= 0.242 |  | 1.01 (0.98,1.05);  p= 0.545 |  |
| CMV reactivation by Day 100 | 3.42 (1.40,8.36);  **p= 0.007** |  |  |  |
| Dad Recipient | 0.61 (0.31,1.23);  p= 0.168 |  | 1.33 (0.36,4.91);  p= 0.666 |  |
| Donor Age | 0.98 (0.96,1.01);  p= 0.128 |  | 1.03 (0.99,1.07);  p= 0.135 |  |
| High RDRI | 1.97 (1.14,3.40);  **p= 0.015** |  | 1.88 (0.88,4.00);  p= 0.102 |  |
| Myeloid Vs Lymphoid/Aplastic Anemia |  |  |  |  |
| Myeloid Vs Lymphoid | 0.45 (0.25,0.83);  **p= 0.011** |  | 0.51 (0.18,1.44);  p= 0.203 |  |
| RIC vs MA Myelo | 0.93 (0.53,1.63);  p= 0.809 |  | 0.83 (0.37,1.85);  p= 0.647 |  |
| Race/Ethnicity Caucasian | 0.92 (0.47,1.82);  p= 0.818 | p=0.956 | 1.14 (0.33,3.93);  p= 0.835 | p=0.978 |
| Race/Ethnicity Other/Multiple Race | 0.91 (0.41,2.00);  p= 0.808 |  | 0.00 (0.00);  p= 0.991 |  |
| Steroid Use During First 100 days | 1.44 (0.81,2.58);  p= 0.217 |  | 0.90 (0.40,1.99);  p= 0.785 |  |

HR, Hazard Ratio, CI, Confidence Interval

Race (Caucasian, African American, Other Minority) was analyzed as a 3 category variable and therefore type 3 p-values are reported. Only 2 patients were characterized as “Other Minority” in the CMV negative group. Therefore, Type 3 p-values specific to this CMV negative category were not reliably assessed.
